# Newly isolated *Pakpunavirus*: efficacy and safety assessment in light of alternative therapies against *P. aeruginosa* skin infections

**DOI:** 10.3389/fmicb.2026.1807725

**Published:** 2026-04-20

**Authors:** Agnieszka Necel, Małgorzata Stasiłojć, Wojciech Wesołowski, Magdalena Narajczyk, Katarzyna Kosznik-Kwaśnicka, Natalia Kaźmierczak, Łukasz Naumiuk, Hanna Loika, Lidia Piechowicz, Anna Żywicka

**Affiliations:** 1 Department of Medical Microbiology, Faculty of Medicine, Medical University of Gdańsk, Gdańsk, Poland; 2 Department of Cell Biology and Immunology, Intercollegiate Faculty of Biotechnology of University of Gdańsk and Medical University of Gdańsk, Gdańsk, Poland; 3 Department of Molecular Biology, Faculty of Biology, University of Gdańsk, Gdańsk, Poland; 4 Bioimaging Laboratory, Faculty of Biology, University of Gdansk, Gdansk, Poland; 5 Department of Clinical Microbiology, Hospital of the Medical University of Gdansk, Gdansk, Poland; 6 Department of Microbiology and Biotechnology, Faculty of Biotechnology and Animal Husbandry, West Pomeranian University of Technology in Szczecin, Szczecin, Poland

**Keywords:** antimicrobial resistance, *Pakpunavirus*, phage therapy, *Pseudomonas aeruginosa*, skin infections

## Abstract

*Pseudomonas aeruginosa* is the major cause of hospital-acquired infections and morbidity and mortality in individuals with burn wounds, due to the emergence of antibiotic resistance. As a result, some scientists are concentrating on research for alternative treatment, with phage therapy being one of the suggestions. However, a thorough description of the phages under consideration for use is necessary to optimize the treatment process. Thus, we show in this paper that the newly isolated phage vB_Pa_AN-12, member of the *Pakpunavirus* genus, is a perfect fit for phage therapy. It can infect several clinical strains of *P. aeruginosa*, including those resistant to multiple antibiotics. It is also able to decrease the viability of host cells strain by 5 logs in 1 h. Furthermore, it does not carry any harmful genes, and has efficient intracellular development with about 100 progeny virions per infected cell. Additionally, it did not affect the viability of cell lines that represented keratinocytes (HaCaT), fibroblasts (BJ), and monocytes (SC). These results suggest that usage of this phage, especially for skin infections, won't cause any side effects resulting from phage-human cell interactions. Nevertheless, given there is a possibility of phage resistance development, the action of isolated phage should be further investigated in combinations with other antimicrobials.

## 1 Introduction

*Pseudomonas aeruginosa* is an opportunistic pathogen that has a high affinity for various types of tissue. It is one of the most common hospital-acquired pathogens, causing over 7% of nosocomial infections (Centers for Disease Control Prevention, 2023). It is mainly responsible for infection of the lungs, skin, urinary tract, and bloodstream. This bacterium may also cause a chronic infection, especially in patients with cystic fibrosis and skin damage. It is estimated that this bacterium is detected in 10%−29.8% of diabetic ulcers and 12.4%−57% of burn wounds (Wood et al., 2023). The research also suggests that it is a major cause of morbidity and mortality among burned patients, as it may be responsible for up to 77% of burn wound fatalities. Additionally, 11%−25% of *P. aeruginosa* hospital strains are multidrug-resistant (MDR), and the resistant to carbapenems are classified by WHO as high-priority pathogens (Kunz Coyne et al., 2022; WHO, 2024). Such a phenomenon is the result of both intrinsic and acquired resistance to antibiotics. *P. aeruginosa* naturally uses different mechanisms for protection, including low outer membrane permeability, efflux pumps (especially Mex-type), and antibiotic-inactivating enzymes (Bassetti et al., 2018). However, it can also acquire multiple resistance mechanisms, such as the production of extended-spectrum β-lactamases (ESBLs) or carbapenemases (De Kievit et al., 2001; Qin et al., 2022; Avakh et al., 2023). In a 2023 report from the European Center for Disease Prevention and Control, the highest resistance was detected to piperacillin-tazobactam (19.3%), fluoroquinolones (18.6%), carbapenems (18.6%), ceftazidime (16.2%), and aminoglycosides (8.9%).

The growing number of infections by *P. aeruginosa* and increasing resistance among the strains resulted in the higher interest of scientists in the development of new antibiotics or alternative therapies. Current propositions include, for example, the use of plant extracts, molecules, nanoparticles, and phage therapy (Sanya et al., 2023). The last one is based on bacteriophages, which are the viruses able to infect only bacterial cells. Additionally, they are usually specific to strains within the same species or family and do not significantly influence the natural microbiota (Divya Ganeshan and Hosseinidoust, 2019). Such activity may reduce the cost of therapy by avoiding the need for probiotic administration. Despite the fact that phages were discovered over 100 years ago, in most countries, the use of these viruses is available only in the form of experimental therapy. However, many papers show the efficient antibacterial activity of phages and that their addition to standard treatment may improve patients' recovery and survival (Alipour-Khezri et al., 2024).

One of the concerns of phage therapy is the different activity of particular phages against host bacteria, the presence of pathogenic genes in their genomes, and the small amount of data about the influence of phage preparations on human cells. Therefore, each phage considered for phage therapy should be described in detail. This includes, e.g., genome composition, host range, and lytic activity.

The aim of our research was to isolate and characterize bacteriophages that could actively infect clinical strains of *P. aeruginosa*, and to assess safety of the phages toward human skin cells, in the context of their planned use, among others, in the treatment of skin infections. In this paper, we present the characterization of a newly isolated phage, vB_Pa_AN-12, active against *P. aeruginosa* strains, including MDR. Furthermore, we supplemented it with an analysis of the effect of phage lysate on human cell lines, which confirmed the potential of vB_Pa_AN-12 to become a support of standard antibiotic therapy.

## 2 Materials and methods

### 2.1 Bacterial strain

All strains used in this study are listed in Supplementary Table 1. For all experiments, *P. aeruginosa* 232 was used as a host to characterize phage vB_Pa_AN-12. For liquid culture, all bacteria were cultivated in LB medium at 37 °C with shaking (150 rpm), while for solid culture, LB was supplemented with 1% agar. All plates were incubated overnight at 37 °C.

### 2.2 Phage isolation

Bacteriophage vB_Pa_AN-12 was isolated from urban sewage collected at the Gdansk Wastewater Treatment Plant in Poland, as described previously with minor modifications (Necel et al., 2020). Briefly, overnight culture of clinical strain *P. aeruginosa* 232 was diluted in 30 mL of LB medium (ratio 1:100), followed by cultivation until optical density (OD_600_) reached 0.15. Then, 0.5 mL of urban sewage was added. After 3 h of cultivation, the mixture was treated with 1:20 chloroform for 15 min and then centrifuged (3,000 × g, 10 min, 4 °C). The obtained supernatant was filtered through 0.22 μm syringe filters (VWR) and serially diluted in TM buffer (10 mM Tris-HCl, 10 mM MgSO_4_, pH 7.2). Each dilution was poured on an LA plate after mixing with 1 mL of host culture and 2 mL of LB soft agar. After overnight incubation, a single plaque was picked with the sterile loop and transferred to the 4 mL of exponentially growing host culture (OD_600_ = 0.15). After 3 h of cultivation, lysate was treated as above until the plaques with the same morphology were visible.

### 2.3 Phage propagation and enumeration

A lysate of the vB_Pa_AN-12 phage was prepared similarly to the isolation process. The virus was added to an exponentially growing host culture (OD_600_ = 0.15) at an MOI of 0.1 and incubated for 3 h. Then, it was treated with 1:20 chloroform and centrifuged (3,000 x g, 10 min, 4 °C). The obtained supernatant was filtered (0.22 μm syringe filters, VWR) and then enumerated using the standard double agar overlay assay (DLA; Ács et al., 2020). Briefly, serial dilutions of lysate were spotted onto host bacteria, *P. aeruginosa* 232, and the titer was determined by the number of phage plaques formed after overnight incubation.

### 2.4 Genome isolation and functional analysis

The genome of phage vB_Pa_AN-12 was isolated, as described previously, using the MasterPure™ Complete DNA and RNA Purification Kit (Epicentre, Madison, U.S.A.; Necel et al., 2020). Isolated DNA was then sequenced by Genomed using Next-Generation Sequencing (NGS) and the MiSeq (Illumina) platform. Raw reads were filtered and quality-controlled using Fastp version 0.23.4. Genomes were assembled using Spades and MetaViralSpades version 3.15.5. Contigs were verified using viralVerify version 1.1. The obtained sequence was annotated using the PhageScope platform (Wang et al., 2024).

### 2.5 Phylogenetic analysis

The analysis of the vB_Pa_AN-12 sequence similarity to other phages was performed using MEGA12 software, based on the complete genome (Kumar et al., 2024). After BLAST analysis, 18 phages with the highest similarity were chosen for comparison. Additionally, sequences were compared to phage from the same class but different family to properly root the phylogenetic tree. A multiple sequence alignment was prepared using the ClustalW algorithm. The tree was generated with the maximum-likelihood method and 1,000 bootstrap replicates.

### 2.6 Microscopy of virions and morphology of phage plaque

To describe the morphology of phage particles, the phage lysate was concentrated using overnight mixing with 10% of PEG8000 (Lab Empire, Rzeszów, Poland). A pellet of phages was obtained by centrifugation (8,000 x g, 10 min, 4 °C) and then resolved in TM buffer (10 mM Tris-HCl, 10 mM MgSO_4_, pH 7.2). Phage particles were then extracted by chloroform and purified by cesium chloride density gradient centrifugation (Kosznik-Kwaśnicka et al., 2024). Virions were negatively stained with 3% uranyl acetate (pH 4.5) for 15 s and then observed under a transmission electron microscope (Tecani Spirit BioTWIN; ThermoFisher Scientific). Plaque morphology was established by pouring the mixture of phage serial dilutions with 1 mL of host overnight culture and 2 mL of LB soft agar on petri dishes containing 25 mL of LA solid medium. Formed plaques were photographed and measured after 24 h of incubation.

### 2.7 Host range

The ability of phage vB_AN-12 to propagate on different strains, then its propagation host *P. aeruginosa* 232, was estimated based on efficacy of plating (Kutter, 2009). To assess it, the phage titer was enumerated using the previously mentioned DLA method, in which 12 clinical strains of *P. aeruginosa*, one *Klebsiella pneumoniae*, three *Escherichia coli*, two *Enterococcus faecalis*, two *Staphylococcus aureus*, and one *Staphylococcus epidermidis* were used in the top agar layer (Supplementary Table 1). The experiment was performed in three replicates.

### 2.8 Kinetic of adsorption

Characterization of the phage's ability to adsorb to the bacterial cell was performed using previously described protocol with small modifications (Topka et al., 2019). Briefly, 60 mL of host culture (OD = 0.3) was centrifuged (3,000 × g, 10 min, 4 °C), and the pellet was washed three times with 10 mL of 0.85% NaCl, then resuspended in 15 mL of LB medium. Next, 1.2 mL of mixture was transferred to the new tube and incubated for 15 min at 37 °C. After infection with phage at MOI = 0.1 (time 0), 100 μL samples were withdrawn at indicated times and centrifuged (6,000 rpm, 10 min, 4 °C). Supernatant containing unabsorbed phages was then enumerated (Section 2.3). The experiment was performed in triplicate, and the values obtained at each time were compared to the titer at time 0, which is considered 100% unabsorbed phages.

### 2.9 One-step growth analysis

To estimate the kinetics of phage progeny, the protocol described earlier was used with modifications (Topka-Bielecka et al., 2020). Shortly, 10 mL of host culture (OD = 0.2) was centrifuged (3,000 x g, 10 min, 4 °C), and bacteria were dissolved in 1 mL of LB medium supplemented with 3 mM NaN_3_. Phage was added at MOI = 0.1 after 5 min incubation at 37 °C. Next, the incubation of the mixture for 10 min at 37 °C was followed by centrifugation and washing in LB containing 3 mM NaN_3_. The final pellet was then resolved in 1 mL of LB medium, and 0.5 mL of phage-infected bacteria was added to 25 mL of LB medium previously heated to 37 °C. Such a mixture was cultivated with shaking (150 rpm) at the same temperature. After 1 min from bacteria addition, a 1 μL sample was added to 999 μL of host overnight culture and mixed with 2 mL of soft LB medium. The mixture was then poured on solid LB medium, and after overnight incubation, the number of infection centers was estimated based on the number of plaques. Additional samples from indicated times were centrifuged with chloroform to release whole progeny and enumerated (Section 2.3). The experiment was done in three replicates, and the burst size was estimated based on the eclipse period, the titer, and the number of infection centers.

### 2.10 Lysis profile assay

The lytic activity of vB_Pa_AN-12 phage was tested using a lysis profile assay, as described previously (Necel et al., 2020). Briefly, the overnight culture of the host was 100-fold diluted in LB medium and cultured until OD = 0.15. The phage lysate was then added at MOIs: 0.01, 0.1, and 1 and cultivated for 3 h. During that time, samples were taken every 30 min and analyzed. One sample was measured at 600 nm (OD_600_) to evaluate changes in optical density. The second sample was serially diluted, and each dilution was poured on LA plates. Based on the number of colonies grown after overnight incubation, the influence of phages on the viability of bacterial cells was assessed (CFU/mL). The last sample was centrifuged with 5% chloroform (5,500 rpm, 5 min), and the supernatant was enumerated to assess the number of phage virions (PFU/mL).

### 2.11 Analysis of viability of human cell lines after treatment with phage lysate

Normal human fibroblasts (BJ, ATCC^®^ CRL-2522™), monocytes (SC, ATCC^®^ CRL-3622™), and keratinocytes (HaCaT, Cytion 300493) were cultured at 37 °C in a humidified atmosphere with 5% CO2 in Eagle's Minimum Essential Medium (EMEM), Iscove's Modified Dulbecco's Medium (IMDM), and Dulbecco's Modified Eagle Medium (DMEM), respectively.

For BJ and HaCaT cells, 1.0 × 10^4^ cells per well were seeded on a 96-well plate and incubated overnight to allow attachment. Afterwards, cells were treated with serial dilutions of phage lysate ranging from 1.0 × 10^5^ to 1.0 × 10^10^ PFU/mL for 24 h, as described before (Kosznik-Kwaśnicka et al., 2023). For SC cells, 2.0 × 10^4^ cells were seeded in a 96-well plate and treated with serial dilutions of phage lysate for 24 h. Cells treated with medium alone served as the negative control, while cells treated with 10% DMSO served as the positive control.

To assess viability, 15 μL of 5 μg/mL MTT (Sigma-Aldrich) solution in PBS was added to each well and incubated for another 2 h. Subsequently, for SC cells, 120 μL of isopropanol containing 0.04 M HCl was added to dissolve the formazan crystals. For BJ and HaCaT cells, the medium was discarded and the wells were washed with PBS. Next, 100 μL of DMSO was added to each well and incubated with shaking for 5 min. Absorbance at 570 nm was measured using a Synergy H1 plate reader (Biokom).

To assess cell membrane integrity, LDH release assay was performed using CytoTox 96 (Promega) according to the manufacturer's protocol. Briefly, after cells were treated with phage lysate as previously described, 50 μL of medium was transferred to a fresh microplate and 50 μL of LDH Substrate Mix was added. For SC cells, the suspension was transferred to a V-bottom plate and centrifuged to aspirate the medium. After 30 min of incubation in the dark, Stop Solution was added to the wells. For the positive control, wells were treated with Lysis Buffer 45 min before the end of the incubation with phages. Absorbance at 490 nm was measured using a Synergy H1 plate reader (Biokom).

## 3 Results

### 3.1 Genomic characterization of vB_Pa_AN-12 and its relatedness

**Figure 1:**
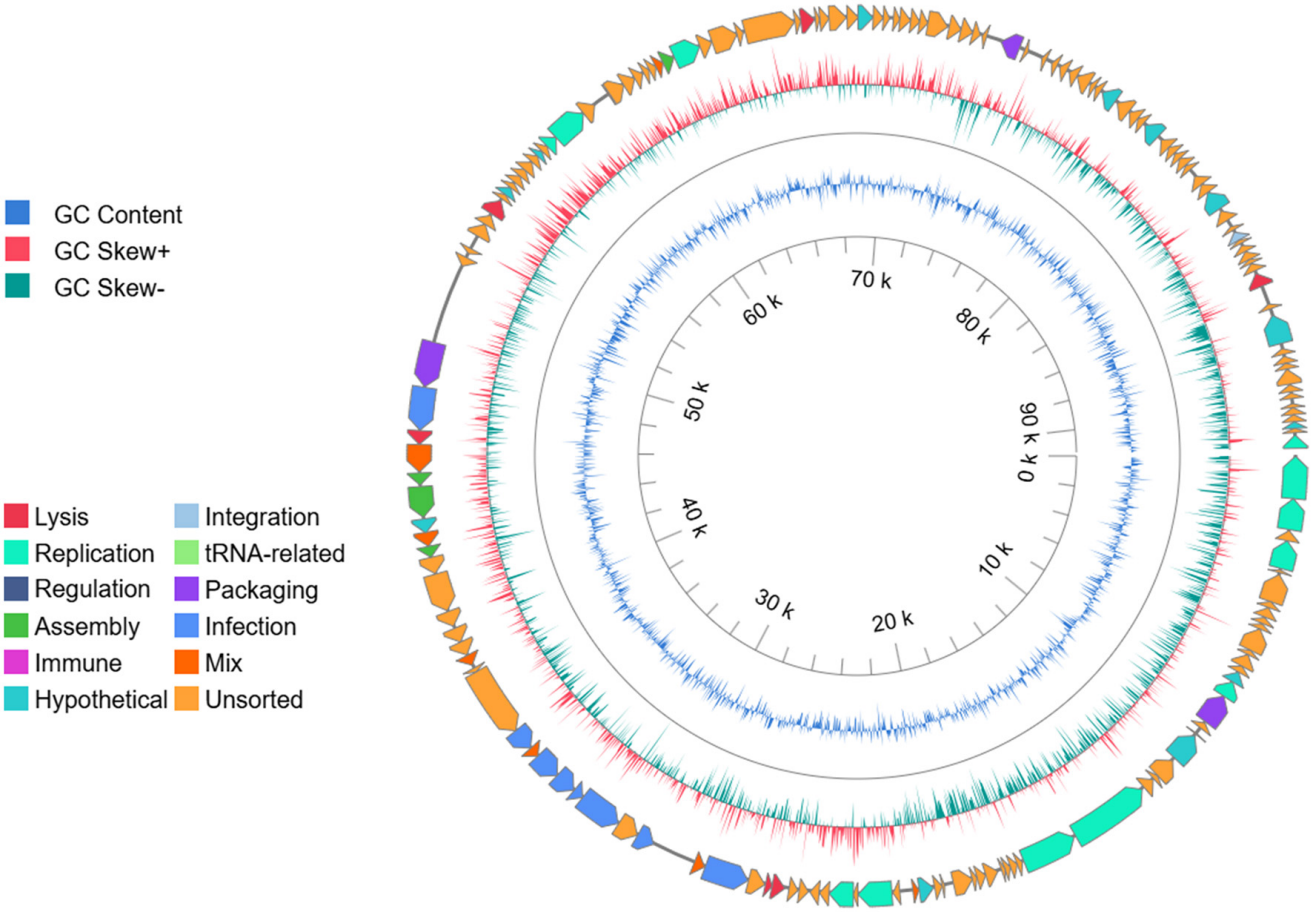
Map of vB_Pa_AN-12 genome created by PhageScope platform. The outer circle describes the potential functionality of hypothetical products. The inner colored circles show the GC parameters.

**Figure 2:**
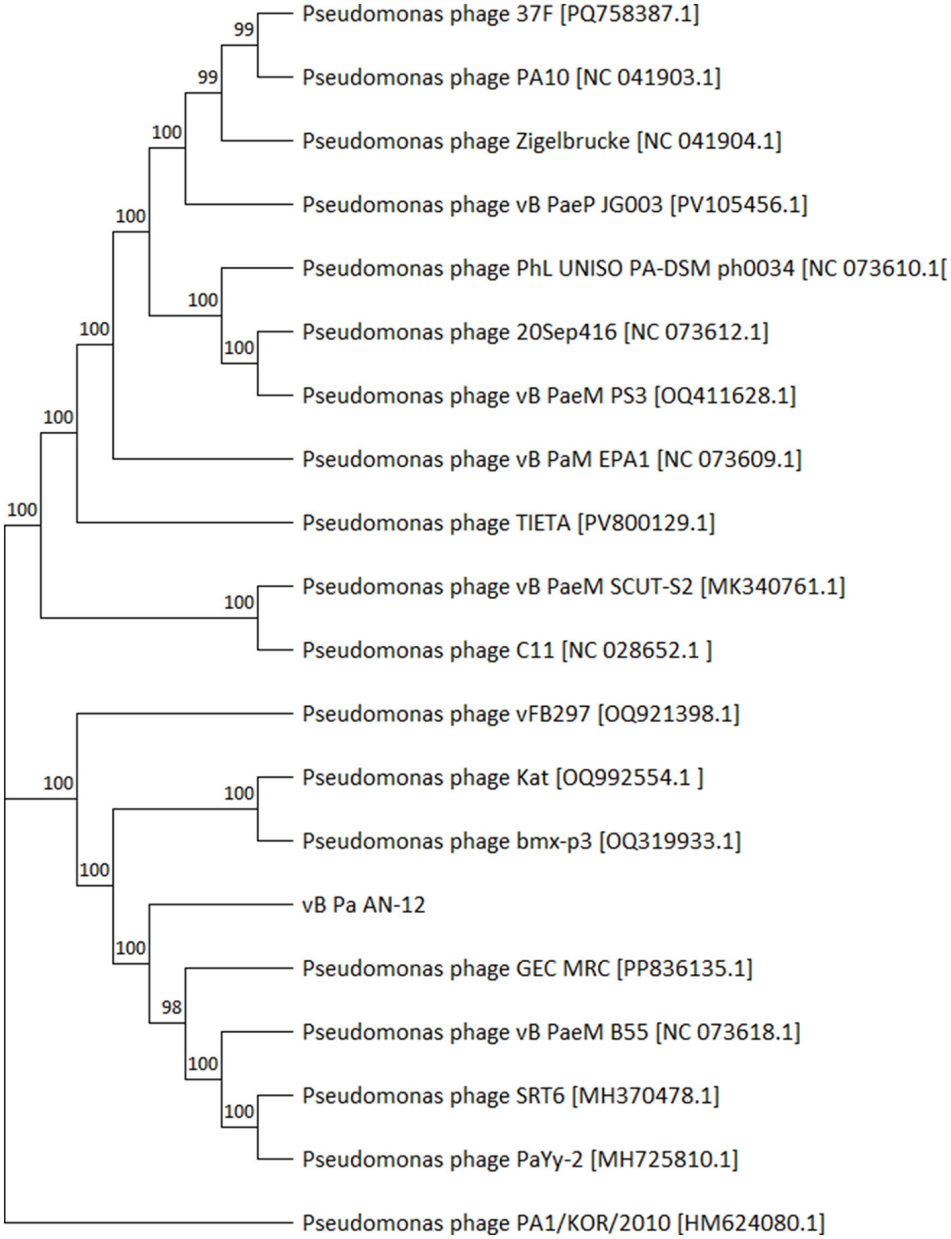
Phylogenetic consensus tree based on the vB_Pa_AN-12 complete genome sequence. The alignment of sequences was performed by using ClustalW. The tree was generated by using MEGA12 and the neighbor-joining method with 1,000 bootstrap replicates, which values are represented at the nodes.

Bioinformatic analysis of the phage genome revealed a sequence with a length of 91,497 bp, the details of which can be found at GenBank (accession number: PX951411). Analysis of the sequence by the PhageScope Platform indicates that it possesses 168 potential genes, where for 55 hypothetical products a possible function was predicted (Figure 1). Most of the predicted products (18) were classified as structural proteins and/or engaged in infection, like tail fiber proteins. According to results, isolated genetic material also possesses 13 genes whose products regulate processes of replication, including the family A DNA polymerase and DNA and RNA ligases. Additionally, six genes encoding enzymes responsible for lysis, like holin or RZ-like spanin, were found. Furthermore, analysis also did not show the presence of any hypothetical products related to the lysogenic cycle or toxin production. Phylogenetic analysis revealed that phage vB_Pa_AN-12 displays significant similarity to the pseudomonas phage GEC_MRC [PP836135.1] (coverage 96%, identity 96.19%). Additionally, the phylogenetic tree showed that this phage belongs to the *Pakpunavirus* genus within the *Vandenendeviridae* family and the *Caudoviricetes* class (Figure 2).

### 3.2 Virion and plaque morphology

**Figure 3:**
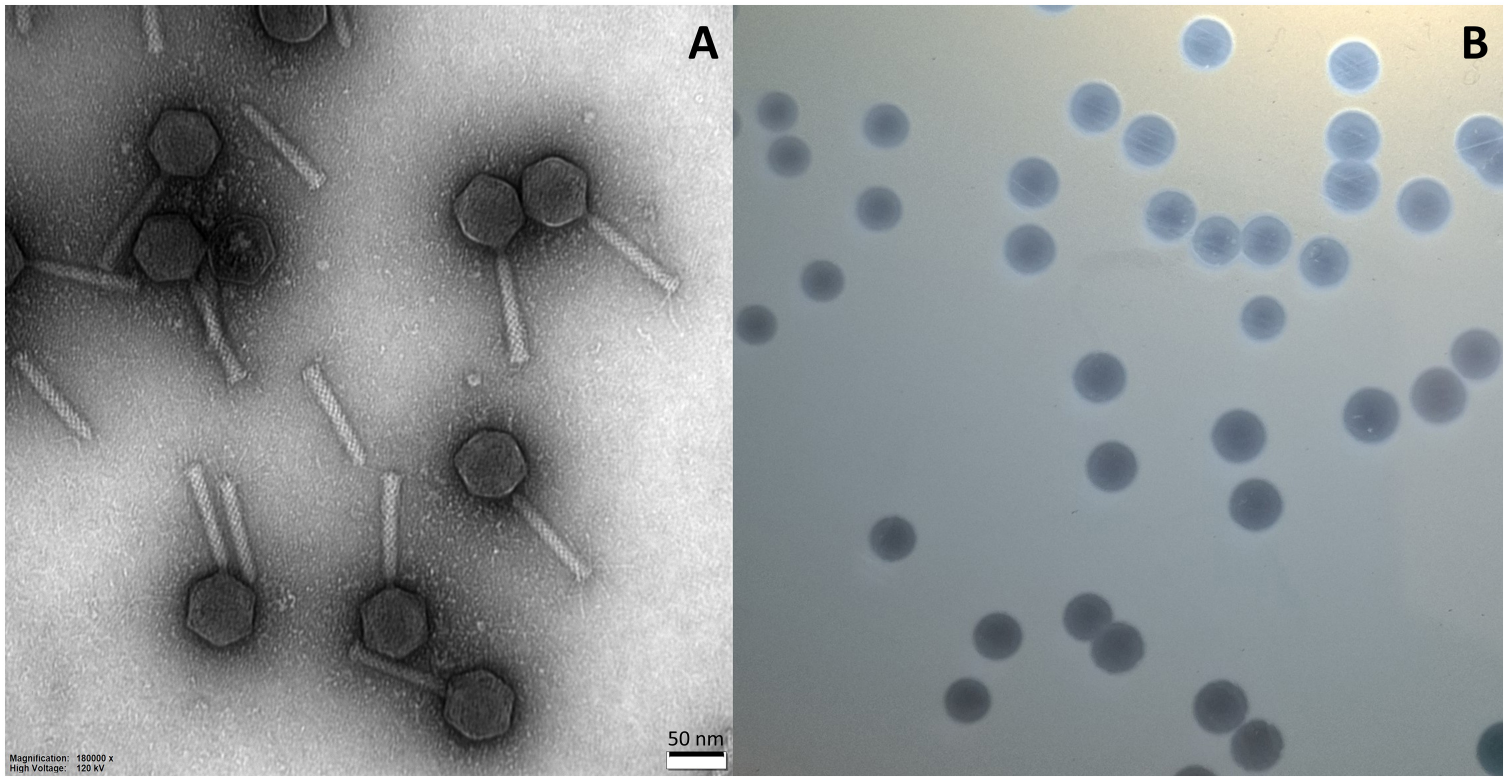
Transmission electron micrograph of vB_Pa_AN-12 phage particles representing morphology of virions **(A)** and photo of plaques formed by this phage on *P. aeruginosa* 232 strain **(B)**.

A TEM analysis of purified particles showed that the isolated phage is myovirus with around 160 nm in length and that it is built with an icosahedral head with a long tail (Figure 3A). In turn, the plaque size, created by phage on isolation host, ranges from 3 to 5 mm and is characterized by the halo zone (Figure 3B).

### 3.3 Host range of the vB_Pa_AN-12 phage

**Figure 4:**
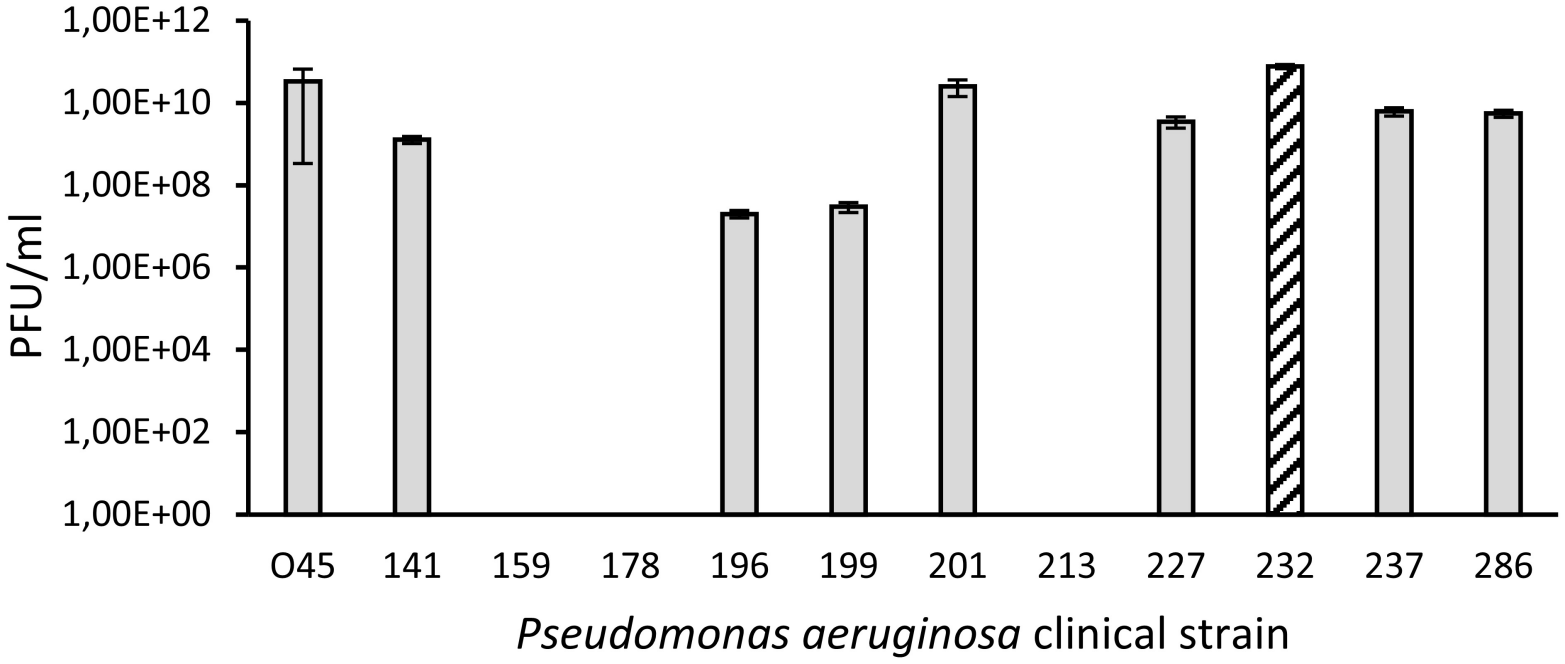
Efficacy of vB_Pa_AN-12 plating on clinical strains of *P. aeruginosa*. Mean values from three independent experiments are shown, with error bars representing SD. The column with lines indicates the host used for isolation and characterization of the phage.

The performed analysis of efficacy of plating revealed that isolated phage is specific for *P. aeruginosa*, since the virus could not propagate in bacteria of other species. However, it was able to lyse 9 from 12 used clinical strains, including *P. aeruginosa* 227, which, according to the antibiogram, is resistant to all tested antibiotics (Supplementary Table S1). Additionally, only for the two strains (*P. aeruginosa* 196 and 199), the titer dropped by more than 2 logs (to 10^7^ PFU/mL), compared to the enumeration on host bacteria (Figure 4).

### 3.4 The life cycle of the vB_Pa_AN-12 phage

**Figure 5:**
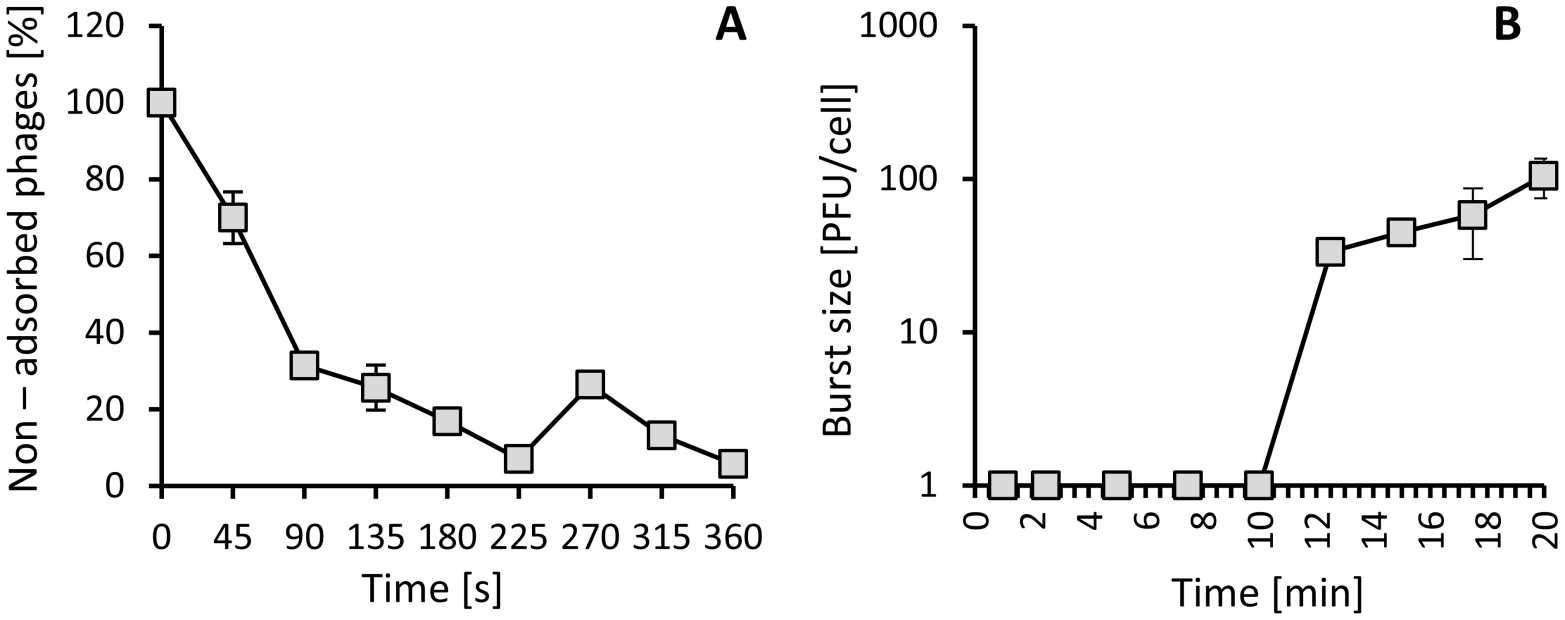
The rate of adsorption of vB_Pa_AN-12 to the *P. aeruginosa* 232 host **(A)** and the curve of progeny production **(B)**. **(A)** Shows the percentage of non-adsorbed virions calculated at the indicated times. **(B)** Shows the number of phage particles produced in a single bacterial cell. The error bars indicate the SD values from three independent experiments.

To describe the life cycle of phage virions, the kinetics of adsorption and progeny production were estimated. According to results, over 80% of viral particles adsorbed to the bacteria in less than 4 min (Figure 5A). We also observed that around 20% of phages desorbed and resorbed to the host. In turn, the one-step growth experiment revealed that this phage starts to produce progeny 10 min after infection (Figure 5B). Moreover, it showed efficient intracellular development, as the number of new viruses in the one 20-min-long life cycle reached about 100 progeny virions per infected cell.

### 3.5 Efficiency of vB_Pa_AN-12 phage to lyse the culture of host bacteria

**Figure 6:**
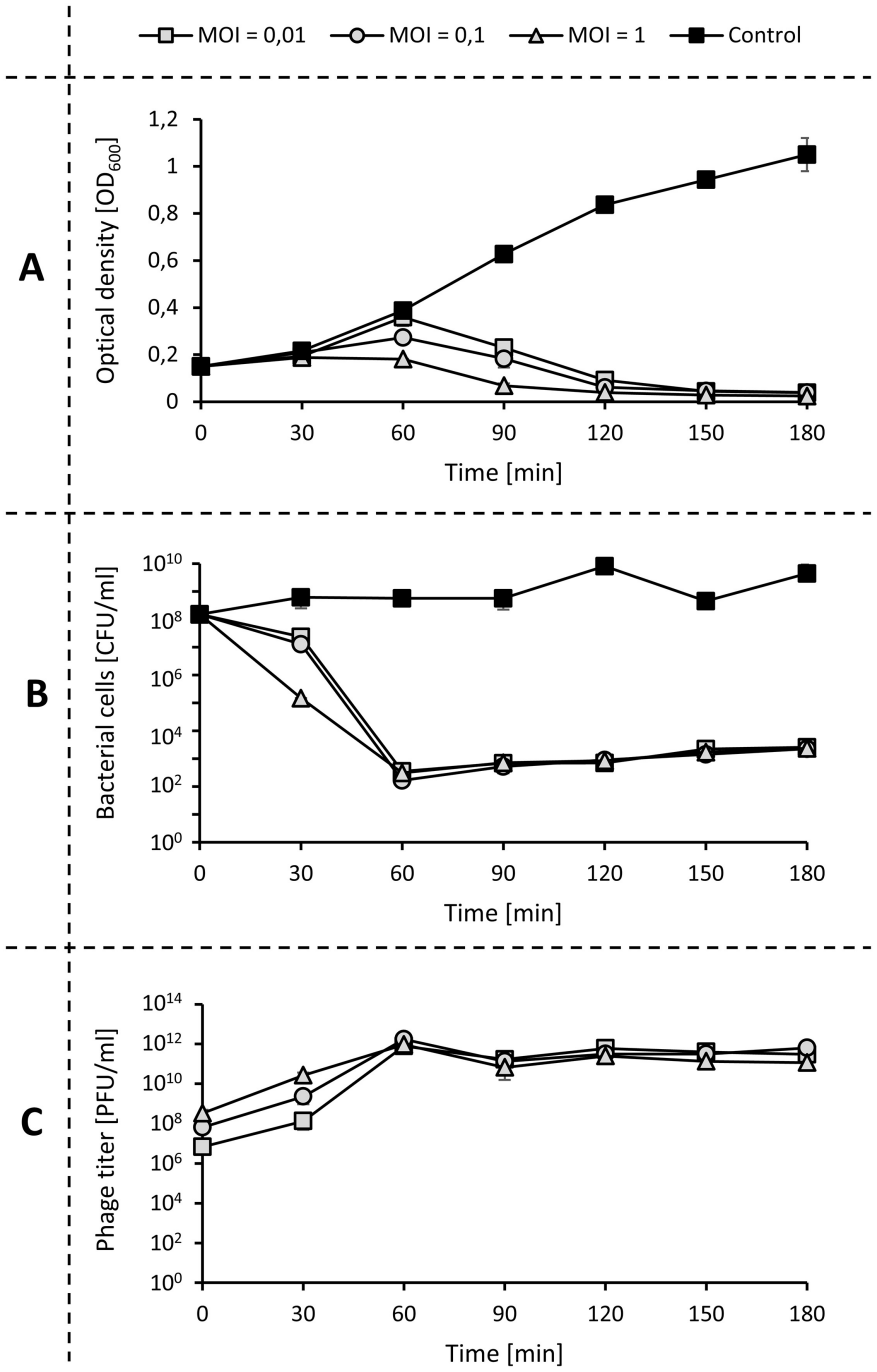
Bacteriophage vB_Pa_AN-12 lysis profile after infection of *P. aeruginosa* 232. Results represent the changes in optical density **(A)**, number of bacterial cells **(B)**, and number of phage particles **(C)**. Plots represent mean values with SD from three experiments, where the cultures of host bacteria without the addition of phage were treated as control.

To estimate the ability of viruses to destroy bacterial cells, three different parameters were monitored: optical density, bacterial cells viability, and number of phages. We observed that phage administration resulted in a significant decrease in optical density after 60 min at all tested MOIs (Figure 6A). Additionally, we observed that the number of viable cells dropped after 30 min of culturing, with a maximum decrease of 5 logs observed at 60 min (Figure 6B). Nevertheless, the used phage did not completely lyse the culture, and the stability of values over the indicates the presence of phage-resistant variants. It is also supported by the lack of phage particle growth observed after 60 min (Figure 6C). What is worth mentioning is that we observed similar behavior at all tested MOIs.

### 3.6 Influence of vB_Pa_AN-12 phage on eukaryotic cells viability and membrane stability

**Figure 7:**
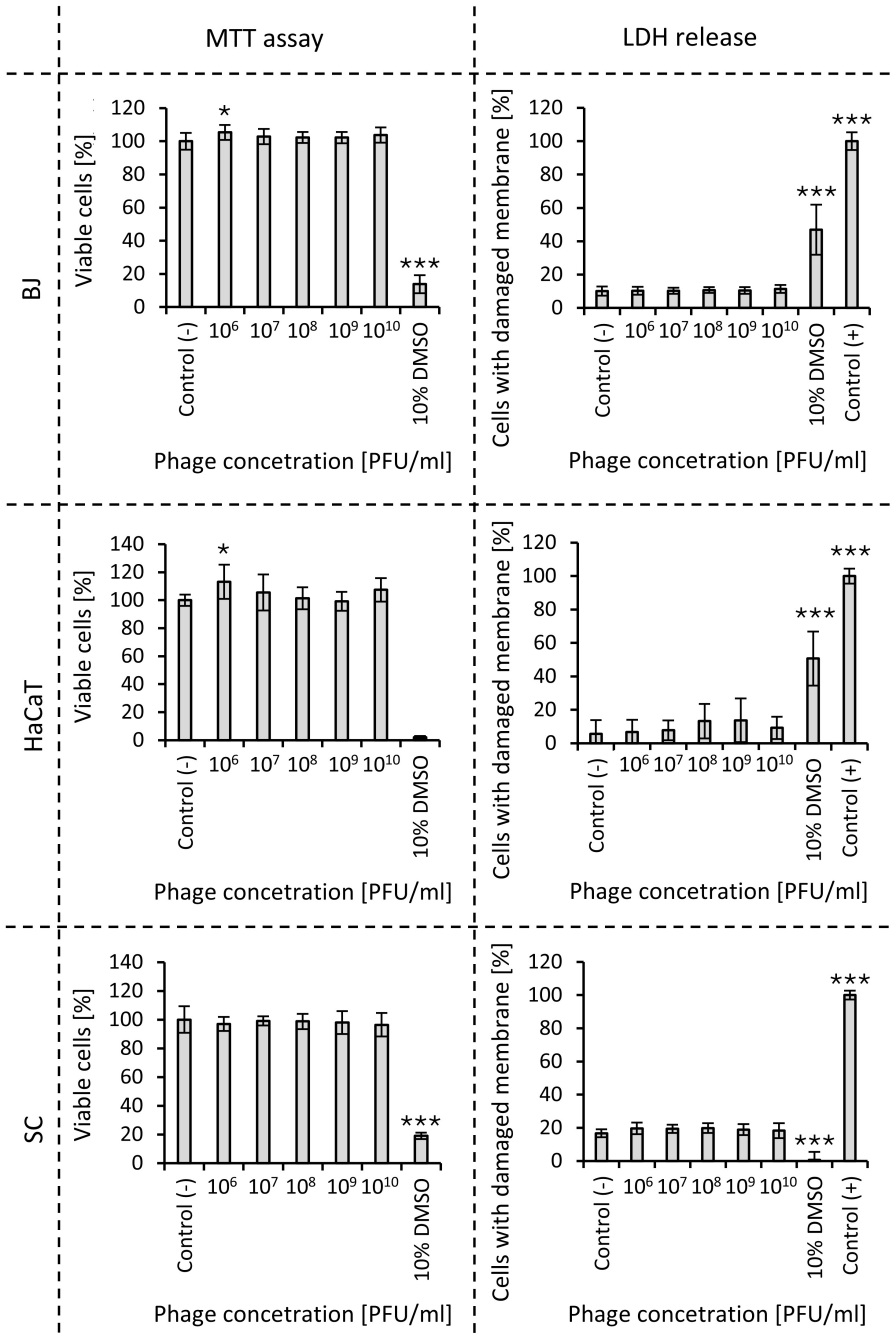
Influence of vB_Pa_AN-12 lysate on BJ, SC, and HaCat cells' viability and cell membrane integrity. For MTT release assay results are presented as a percentage of measures compared to non-treated cells (negative control). The plots showing the membrane stability represent the percentage of released LDH compared to samples treated with lysis buffer (positive control). Statistical analysis was performed using one-way ANOVA with comparison of samples treated with phage to negative control, and Tukey's *post-hoc* test (*0.01 < *p* < 0.05; **0.005 < *p* < 0.01; ***p < 0.005). ^**^statistical significance.

In order to assess the possibility of using vB_Pa_AN-12 in the treatment of skin infection caused by *P. areuginosa*, the safety of phage lysate was estimated using fibroblasts (BJ), monocytes (SC), and keratinocytes (HaCaT; Figure 7). We observed that 24 h incubation of phages with mentioned cell lines did not influence negatively the metabolic activity of any cell line, when tested using MTT assay. Additionally, LDH release assay revealed no statistically significant changes in membrane's stability. Importantly, the lack of toxicity was observed across all tested phage concentrations.

## 4 Discussion

The currently observed increase in pathogens' resistance to antibiotics has become one of the main therapeutic challenges in medicine. In the 2016 report on antimicrobial resistance by O'Neill, it is estimated that by 2050, infections caused by MDR pathogens will take 10 million lives a year and cost 100 trillion USD (O'Neill, 2016). Therefore, many health organizations started to publish materials about the proper use of antibiotics and the prevention of bacteria spreading to slow down the growth of cases. These actions should result in more well-thought-out therapies, thereby reducing selective pressure on resistant strains. However, such actions in the era of overloaded healthcare facilities, delayed treatment, and antibiotic overuse may not be enough to erase antimicrobial resistance from the list of medicine's main issues. Therefore, there are currently many approaches to supply or replace the standard antibiotic-based therapy (Elshobary et al., 2025).

One of the proposed methods to fight drug-resistant pathogens is phage therapy, which, in the case of *P. aeruginosa*, is actively researched (Pires et al., 2015; Nawaz et al., 2024; Chan et al., 2025). However, none of the developed treatments are formally established or approved for incorporation into routine clinical practice. The reason for this may be, e.g., the limited quantity of information about the viruses used and their influence on eukaryotic cells. Therefore, the presented specific characterization of the vB_Pa_AN-12 phage may be one of the next steps to establish the potential of phage therapy. It is noted that bacteriophages considered for use in phage therapy should not have transduction potential or carry toxin genes (Hyman, 2019). These are detected in lysogenic phages, whose usage may result in the decrease of therapeutic outcomes and increase of pathogenic genes spreading. Nevertheless, temperate phages are also considered to be used against MDR bacteria, but mostly after genetic engineering leading to modulation of bacterial host virulence or sensitivity for therapeutics (Cook and Hynes, 2025). We have shown that phage vB_Pa_AN-12 lacks any known genes responsible for toxin production, antimicrobial resistance, or the lysogenic life cycle. Thus, usage of isolated virus does not carry the risk of antibiotic resistance spreading through the transfer of responsible genes to other strains. The genetic data revealed also that the isolated phage belongs to the *Pakpunavirus* genus, which corresponds with the morphology of the virions (Essoh et al., 2015; Suchithra et al., 2023).

During development study, we observed that around 20% of particles create reversible bonding with the receptor at the first contact. After this, the virus searches for the proper receptor to attach to and infect the host bacteria (Bertozzi Silva et al., 2016). The lytic development of vB_Pa_AN-12 was confirmed by the 20 min long life cycle with the creation of over 100 new particles during development in *P. aeruginosa* 232. Such effectiveness in multiplication is probably due to the presence of genes encoding polymerase or ligases, which allow phage to replicate without dependence on the host enzymes (Morcinek-Orłowska et al., 2022). The phage was also active against the used host at all tested MOIs and was able to decrease the number of living bacterial cells by 5 logs in 60 min. However, we observed a difference between time curves of optical density and bacterial viability, which might be the result of continued lytic activity of phages on plates during overnight incubation. Therefore, the number of living cells at the early stage could be higher than the presented values. Such a phenomenon indicates that in phage therapy, the time of treatment might be important to obtain optimal efficiency. This might be especially helpful when combined with other antimicrobials or for providing a time window for the delayed antibiotic treatment (Li et al., 2020; Wang et al., 2025). Additionally, analysis of vB_Pa_AN-12 lytic activity revealed that this phage could not kill all the bacteria in culture. This suggests that bacteria that survived the treatment may have developed resistance to the used phage, which occurrence is commonly observed in virus-treated pathogens. Nevertheless, developed resistance often negatively influences the pathogenic activity of bacteria and leads to changes in sensitivity to other antimicrobials. Chan et al. showed that phage resistance of bacteria to the used OMKO1 phage resulted in an increased sensitivity of the host to ceftazidime, ciprofloxacin, tetracycline, and erythromycin (Chan et al., 2016). The most common mechanisms responsible for such phenomenon are modification or loss of surface receptors. Thus, it might influence not only the attachment of the previously used phage but also to other phages targeting the same receptor. However, there are many approaches to combat the phage resistance occurrence. These rely on the use of phages targeting virulence determinants, engagement of phages that recognize multiple receptors, or application of phages in combination with other viruses or antibiotics. The use of a phage cocktail may also increase the number of strains sensitive to phage treatment, which in the case of vB_Pa_AN-12 was limited to 75% of all tested clinical *P. aeruginosa* (Chen et al., 2024). Moreover, some scientists propose the use of phage-encoded enzymes to control bacteria, as indicated by the presence of holin genes in the vB_AN-12 genome and the halo zone observed in the formed plaques. The advantages of such enzymes over whole phages usually include a broader host range, a lower likelihood of inducing resistance, and easier management (Zhang et al., 2025). Therefore, not only might whole particles of isolated phage be useful for fighting *P. aeruginosa* strains, but also their enzymes alone could serve as therapeutics in the future.

Since phages can be taken up by eukaryotic cells through transcytosis, the influence of phages on their behavior is also one of the currently researched topics (Nguyen et al., 2017). Bearing in mind the high prevalence of *P. aeruginosa* in skin infections, we decided to test the influence of phage lysate on three human cell lines engaged in the skin defense system (Sharma and Kishen, 2025). Used lines represent fibroblasts (BJ), monocytes (SC), and keratinocytes (HaCaT). Neither the viability of human cells nor their membrane integrity was significantly influenced by any concentration of phages. This implies that phage vB_Pa_AN-12 does not interact with chosen cell lines and won't cause a negative effect during the treatment. However, such conclusions should be confirmed by testing different cell lines and mammalian models.

## 5 Conclusions

Based on obtained results, we assume that the bacteriophage vB_Pa_AN-12 is characterized with features that fit phage therapy. It can actively reduce the bacterial load of clinical *P. aeruginosa* strains and does not affect the viability of eukaryotic cells. However, to prevent the development of phage resistance, it should be used in combination with other antimicrobials. To achieve optimal therapeutic outcomes, such combinations should be tested to determine the highest synergistic effects and the optimal dosing time. Nevertheless, to confirm the therapeutic potential of isolated phage, further research is needed, particularly through experiments in animal models.

## Data Availability

The datasets presented in this study can be found in online repositories. The names of the repository/repositories and accession number(s) can be found in the article/Supplementary material. Specifically, the data are available in Bridge of Knowledge under DOI 10.34808/a7pt-9k64, and the NCBI GenBank under accession number PX951411.
