# Supplementary material for: Newly isolated Pakpunavirus: efficacy and safety assessment in light of alternative therapies against P. aeruginosa skin infections
Source: Front Microbiol. 2026 Apr 20;17:1807725. doi: 10.3389/fmicb.2026.1807725 (PMC13136285; doi:10.3389/fmicb.2026.1807725)
Supplement: Supplementary file 1 [file Table_1.docx]

Supplementary Material

# Supplementary Table 1. List of bacterial strains used in experiments.

| Bacterial strain | | Source | Antibiotic resistance |
| --- | --- | --- | --- |
| Clinical *P. aeruginosa* | O45 | Laboratory of Clinical Microbiology, University Clinical Center,  Gdańsk, Poland | **Intermediate:** piperacillin/tazobactam, ceftazidime, cefepime, and ciprofloxacin; |
|  | 141 |  | **Intermediate:** piperacillin/tazobactam, ceftazidime, cefepime, and ciprofloxacin; |
|  | 159 |  | NA |
|  | 178 |  | NA |
|  | 196 |  | NA |
|  | 199 |  | **Resistance:** piperacillin/tazobactam, ceftazidime;  **Intermediate:** cefepime, cefoperazone/sulbactam, meropenem, and ciprofloxacin; |
|  | 201 |  | **Intermediate:** piperacillin/tazobactam, ceftazidime, cefepime, and ciprofloxacin; |
|  | 213 |  | NA |
|  | 227 |  | **Resistance:** piperacillin/tazobactam, ceftazidime, cefepime, cefoperazone/sulbactam, meropenem, amikacin and ciprofloxacin; |
|  | 232 |  | **Intermediate:** piperacillin/tazobactam, ceftazidime, cefepime, and ciprofloxacin; |
|  | 237 |  | NA |
|  | 286 |  | NA |
| *Klebsiella pneumoniae* ATCC^®^ 700603 | | Department of Medical Microbiology, Medical University of Gdańsk  (originally from ATCC^®^) | **Resistance:** ampicillin, aztreonam, cefoxitin, cefpodoxime, ceftazidime, chloramphenicol, piperacillin, and tetracycline;  **Intermediate:** ceftriaxone and gentamicin; (Rasheed et al., 2000) |
| *Escherichia coli*  ATCC^®^ 11755 | |  | NA |
| *Escherichia coli*  ATCC^®^ 35218 | |  | Contains plasmid-encoded TEM-1 β-lactamase (non-ESBL) |
| *Escherichia coli*  ATCC^®^ 25922 | |  | **Resistance:** Sulfamethoxazole/trimethoprim, cefotaxime, cefixime, ceftriaxone, and chloramphenicol;  **Intermediate:** ampicillin, gentamycin, erythromycin; (Mursyida et al., 2023) |
| *Enterococcus faecalis* ATCC^®^ 51299 | |  | **Resistance:** vancomycin |
| *Enterococcus faecalis* ATCC^®^ 29212 | |  | NA |
| *Staphylococcus aureus* ATCC^®^ 25923 | |  | NA |
| *Staphylococcus aureus* ATCC^®^ 29213 | |  | NA |
| *Staphylococcus epidermidis* ATCC^®^ 14990 | |  | NA |
